# Musculoskeletal infections caused by streptococcus infantarius – a case series and review of literature

**DOI:** 10.1007/s00264-025-06487-3

**Published:** 2025-03-13

**Authors:** Alberto Alfieri Zellner, Julian Voss, Alexander Franz, Jonas Roos, Gunnar Thorben Rembert Hischebeth, Ernst Molitor, Frank Sebastian Fröschen

**Affiliations:** 1 University Hospital of Bonn, Department of Orthopedics and Trauma Surgery, Bonn, Germany; 2 University Hospital of Bonn, Institute of Medical Microbiology, Immunology and Parasitology, Bonn, Germany

**Keywords:** PJI, Streptococci, Streptococcus infantarius, Musculoskeletal infection, Antibiotic susceptibility

## Abstract

**Purpose:**

The full spectrum of diseases caused by *S. infantarius* remains poorly understood, particularly its role in musculoskeletal infections.

**Methods:**

A retrospective study was conducted from January 2008 to May 2024. Patients with bacterial infections and detection of *S. infantarius* in at least one tissue sample, fluid sample, or blood cultures were included. Follow-up controls in patients with musculoskeletal infection were performed.

**Results:**

*S. infantarius* could be identified in at least one sample (blood cultures, wound fluid, wound swab, bile, tissue or urine sample) of 72 patients. 33 were considered clinically relevant with symptomatic infections (63.4 ± 21.1 years; positive samples: 1.39 ± 0.86; total number of samples: 2.7 ± 1.76). Non-muskuloskeletal infections (*n* = 29; 61.1 ± 21.5 years; positive samples: 1.28 ± 0.59) included a variety of different infections (sepsis (*n* = 11), abdominal/gastrointestinal/urogenital infections (*n* = 16), soft tissue infections (*n* = 2)). Four patients with musculoskeletal *S. infantarius* infection (positive samples: 2.25 ± 1.89; diagnosis: acute PJI, spondylodiscitis, chronic PJI and postoperative spinal wound infection) required surgical and/or antimicrobial treatment. Follow-up after musculoskeletal infection varied between 10 and 60 months. Antibiotic susceptibility testing displayed a sensitivity to Penicillin in all isolates. No patient had a recurrent positive sample/infection with *S. infantarius*.

**Conclusions:**

This study describes musculoskeletal infections caused by *S. infantarius*, highlighting its possible relevance as pathogen in orthopedic infections. The findings underscore the importance of recognizing and appropriately treating *S. infantarius*. In case of penicillin allergy, clindamycin shows to be an effective alternative treatment.

## Introduction

The *S. infantarius*, belongs to the *Streptococcus bovis* group and was described for the first time in the year 1997 [1, 2]. However, clinically relevant infections caused by this rare pathogen may have appeared earlier, without knowing that it was a subspecies. *S. infantarius* was further described in 2001 by molecular method (16 S rRNA gene sequence analysis [MicroSeq]) and is considered a member of the *Streptococcus bovis/Streptococcus equinus* complex [3]. In 2003, its taxonomy was further reappraised by Schlegel et al. [4]. The *Streptococcus bovis/Streptococcus equinus* complex comprises a group of bacteria that share many genetic similarities, are alpha or nonhaemolytic streptococci and mostly express Lancefield group D antigens [5, 6]. Morphologically, *S. infantarius* is characterized as a Gram-positive, non-motile, non-spore-forming coccus. Under the microscope, it appears as spherical or ovoid cell arranged in pairs or short chains. A formation typical for streptococci. *S. infantarius* is primarily associated with African dairy production and is only rarely seen as a human pathogen [7–9]. As a pathogen it has been detected in several clinical settings, including bacteremia, endocarditis while an association with colorectal cancer is suspected [5]. However, due to its rarity, the full spectrum of diseases caused by *S. infantarius* is not yet fully understood. Studies have suggested a potential link between infection with certain strains of the *S. bovis* group and the development of colorectal cancer, although the underlying exact mechanisms remains unclear [10–14]. Furthermore, cases of infective endocarditis caused by *S. infantarius* have been reported, highlighting its pathogenic potential [15]. The microbiological identification of *S. infantarius* can be challenging, suggesting that infections caused by *S. infantarius* might occur unnoticed as routine diagnostics might not be able to detect *S. infantarius* [16].

To our knowledge this is the first study analyzing musculoskeletal and periprosthetic joint infections (PJI) caused by *S. infantarius*. Musculoskeletal infections including PJI can lead to significant morbidity of the patients, necessitating complex medical and surgical interventions, and are a major cause of e.g. implant failure in the field of prosthetic surgery.

In general, musculoskeletal infections can be caused by a variety of microorganisms, including bacteria and fungi. Among bacterial pathogens, *Staphylococcus aureus* and coagulase-negative staphylococci are the most frequently detected [17].

However, streptococci, particularly *Streptococcus agalactiae* or *Streptococcus pyogenes* play a significant role in musculoskeletal infections [18].

Musculoskeletal infections caused by streptococci, might be characterized by an (sub-) acute onset of symptoms with severe local and systemic inflammation, requiring a fast diagnosis and treatment for best possible outcomes. Better understanding of the pathogens responsible for musculoskeletal infections, is crucial for developing effective treatment strategies. This includes evaluation of rare pathogens such as *S. infantarius*.

We aim to evaluate musculoskeletal infections caused by *S. infantarius* by presenting a case series of musculoskeletal infections found in our department and all non-orthopedic cases with detection of *S. infantarius* in our university hospital.

## Materials and methods

We conducted a retrospective study at the Institute of Medical Microbiology, Immunology and Parasitology and the Department of Orthopedics and Trauma Surgery January 1st, 2008, to May 31st, 2024. Ethical approval was obtained from the institutional review board (IRB) (No. 2024/164) and was conducted following the ethical standards of the 1964 Declaration of Helsinki and its subsequent amendments. Since this was a retrospective study using anonymized data, informed consent was waived by the IRB.

Inclusion criteria were presence of an infection with need for treatment and with detection of *S. infantarius* from intraoperative tissue samples, fluid or blood cultures. For confirmation of a clinically relevant infection all cases were evaluated by four independent observers. Exclusion criteria were missing need for treatment/absence of symptoms.

For better description of the included patients, we recorded patient demographics such as weight, comorbidities, antimicrobial and/or surgical therapy. For patients with musculoskeletal infections an evaluation of the follow-up after discharge was performed. After a musculoskeletal infection, routine follow-up visits are performed at 6 weeks, 6 months, 12 months and thereafter in 12 month intervals.

### Microbiological workup

Tissue specimens were collected under sterile conditions and send to the microbiological laboratory in sterile tubes. After homogenization intraoperative collected tissue specimen were plated onto Columbia agar with 5% sheep blood (Becton & Dickinson, Heidelberg, Germany), Mac Conkey agar (Becton & Dickinson, Heidelberg, Germany), chocolate agar (Becton & Dickinson, Heidelberg, Germany), sabouraud agar (Becton & Dickinson, Heidelberg, Germany) and in enrichement thioglycolate bullion (Becton & Dickinson, Heidelberg, Germany). Schaedler and KV agar plates (Becton & Dickinson) for anaerobic cultures were similarly striked with homogenizated tissue specimen. Cultures were grown at 5% CO2 and 35 °C. All specimens were cultured for 14 days.

Wound swabs were rolled directly onto the culture plates (see above) and then striked in typical manner. Depending on the depth of the wound, anaerobic cultures were also created.

Joint aspirates or sonication fluid were cultured like the tissue samples but also inoculated in PEDS blood culture bottles (Becton & Dickinson) and incubated for 14 days in a Bactec FX blood culture system (Becton & Dickinson).

Urine cultures were performed according to laboratory standard. In brief, Columbia, Mac Conkey, Candida select (Becton & Dickinson, Heidelberg, Germany) were inoculated with 10 µl urine, striked and cultured for 2 days in an incubator at 35 °C. Agar plates were checked for bacterial or fungal growth every 24 h. If suspicious pathogenic growth was detected, an identification was carried out and an antibiogram was prepared.

Species identification was done with mass spectrometry via matrix-assisted laser desorption ionization time of flight analysis (Vitek-MS MALDI-TOF, bioMérieux, Nürtingen, Germany). Additionally, the antimicrobial susceptibility testing was performed with an automated antimicrobial susceptibility testing system, Vitek2 (bioMerieux).

### Statistical analysis

Data were collected in Microsoft Excel 2024 (Microsoft Corporation, Richmond, VA, USA). Statistical analysis was carried out with SPSS statistics 28 for Windows (SPSS, Inc. an IBM company, Chicago, IL, USA). Descriptive statistics, including median value and range were calculated. Data are given as median and ranges, if not indicated otherwise.

## Results

A total of 72 interdisciplinary cases with detection of *S. infantarius* in tissue samples, fluid samples, wound swabs, sonications, urine and blood cultures could be identified between January 1st, 2008 and May 31st, 2024. Seven of those were associated with an infection in the musculoskeletal system. Of the remaining 65 interdisciplinary cases we were able to identify 29 cases that could be classified as infection and received a surgical and/or antimicrobial treatment (Table 1). The remaining 36 cases had either a polymicrobial infection or solely detection of *S. infantarius* in one sample and only after enrichment or were classified as non-clinically relevant as patients did not display symptoms. In this context we would like to outline detection of *S. infantarius* in urine samples. Out of 14 detections only one case could be classified of clinical relevance. This patient had a symptomatic urinary tract infection and had a bacterial concentration of more > 10^5^ colony forming units/ml. In those remaining patients with polymicrobial infections, an interdisciplinary retrospective evaluation could not confirm a clinically relevant *S. infantarius* infection.

In total, 29 patients were classified as non-musculoskeletal infection with *S. infantarius*, while 4 patients with suffered under a musculoskeletal infection (n_total_=33; Age: 63.4 ± 21.1; number of samples with detection of *S. infantarius*: 1.39 ± 0.89; total number of samles: 2.7 ± 1.76). In patients with a non-musculoskeletal infection (age: 61.1 ± 21.5 years), detection of *S. infantarius* was possible in 1.28 ± 0.59 samples (total number of samples: 2.34 ± 1.54) while in patients with musculoskeletal infection (*n* = 4) *S. infantarius* could be detected in 2.25 ± 1.89 samples (total number of samples: 5.25 ± 0.96). There was no case with recurrent microbiological detection of *S. infantarius*.

**Table 1:** Non-musculoskeletal infections with *S. infantarius* by culture type (M = male; F = female; FAP: Familial adenomatous Polyposis)

Nr. of Cases	Sex	Culture Type	Diagnosis	Treatment	Outcome
11	M (*n* = 9) F (*n* = 2)	blood cultures	sepsis (*n* = 2), uro-sepsis (*n* = 3), pneumonia sepsis (*n* = 3) endocarditis (*n* = 1), cholangitis sepsis (*n* = 2)	Surgical: *n* = 1	Treatment success *n* = 5
				Antibiotics: *n* = 11	treatment failure *n* = 6
6	M (*n* = 3) F (*n* = 3)	wound fluid aspiration	lymphocele (*n* = 1), FAP (*n* = 1), abdominal abscess (*n* = 1), bartholinabscess (*n* = 1), necrotizing pancreatitis (*n* = 1), sigmaperforation (*n* = 1)	Surgical: *n* = 6	Treatment success *n* = 6
				Antibiotics: *n* = 6	treatment failure *n* = 0
5	M (*n* = 3) F (*n* = 2)	wound swabs	abdominal wall abscess (*n* = 2), perforated sigma diverticulitis (*n* = 1) pancreas carcinoma (*n* = 1), colon fistula (*n* = 1)	Surgical: *n* = 5	Treatment success *n* = 4
				Antibiotics: *n* = 5	treatment failure *n* = 1
5	M (*n* = 3) F (*n* = 2)	bile	cholezistolithiasis (*n* = 1), mechanical ileus (*n* = 1), cholangitis (*n* = 3)	Surgical: *n* = 1	Treatment success *n* = 4
				Antibiotics: *n* = 5	treatment failure *n* = 1
1	M (*n* = 1)	biopsy, tissue sample	gastritis (*n* = 1)	Surgical: *n* = 0	Treatment success *n* = 1
				Antibiotics: *n* = 1	treatment failure *n* = 0
1	M (*n* = 0) F (*n* = 1)	urine sample	cystitis (*n* = 1)	Surgical: *n* = 0	Treatment success *n* = 1
				Antibiotics: *n* = 1	treatment failure *n* = 0
29	M (*n* = 19) F (*n* = 10)			Surgical: *n* = 13	Treatment success *n* = 19
				Antibiotics: *n* = 29	treatment failure *n* = 10

Musculoskeletal infections with detection of *S. infantarius* are displayed in Table 2.

**Table 2:** Musculoskeletal infections with detection of *S. infantarius* (PJI: periprosthetic joint infections; DAIR: debridement, antibiotics, irrigation and implant retention; I.v.: intravenous; treatment success is defined as eradiation of the infection)

Case Nr.	Age	Sex	Diagnosis	Culture Type	Treatment	Antibiotics	Outcome
1	80	M	Acute PJI of the Knee	Sonication tissue samples	DAIR	i.v.: Penicillin G oral: Amoxicillin	Treatment success
2	84	M	Spondylodiscitis	preoperative blood cultures	surgical	i.v.: Vancomycin, Rifampicin oral: Amoxicillin, Clindamycin	Treatment success
3	76	F	Chronic PJI of the Hip	sonication of explanted prosthetic material	hip resection arthroplasty	i.v.: Unacid, Ceftazidim oral: /	Treatment success
4	79	M	Postoperative wound infection after Spondylodiscitis	central blood cultures	conservative	i.v.: Penicillin G oral: Amoxicillin	Treatment success

In summary, detection of *S. infantarius* was possible in seven cases in the Department for Orthopedics and Trauma Surgery. Four were classified as infection and underwent appropriate treatment. Three cases were regarded as contamination after review of all findings (e.g. histopathological evaluation) and were not specifically targeted.

Table 3 below displays the results of the antibiotic susceptibility testing in the 43 samples taken from the 33 clinically relevant cases. *S. infantarius* displayed a susceptibility to penicillin in of all cases. No isolate displayed a resistance to Penicillin. Susceptibility rate to Clindamycin was 85.7%. 14.3% of the isolates displayed a resistance to clindamycin.

No isolate displayed a resistance against Vancomycin.

**Table 3:** Results of the antibiotic susceptibility testing in *S. infantarius* of all 43 Microbiological samples from all 33 clinically relevant interdisciplinary cases. The susceptibility testing was performed according to EUCAST (breakpoint tables V 14.0) (r = resistant; s = susceptible)

Pathogen	Penicillin G		Clindamycin		Vancomycin	
	s	r	s	r	s	r
*S. infantarius*	43	0	36	6	43	0

### Patient 1

80-year-old male patient, who presented initially because of persisting symptoms in his right knee. Previously, the patient had undergone total knee arthroplasty (TKA) in a foreign hospital because of osteoarthritis followed by a single-stage exchange to a constrained knee prosthesis. Three years later he had spontaneous symptoms in his right knee with pain, redness, swelling and fever of up to 40 °C and was admitted to our hospital. After diagnostics had been performed (blood samples, joint aspiration with cell count) an acute late periprosthetic joint infection of inlying arthroplasty was diagnosed. Surgical treatment consisting of arthrotomy, debridement, lavage exchange of mobile bearing of the TKA and subsequent antibiotic therapy (DAIR procedure) was performed. Detection of *S. infantarius* was possible from all (5/5) intraoperative specimen taken, including sonication as well as in the preoperative blood cultures. The histopathological examination revealed a type II membrane according to Krenn and Morawietz substantiating the diagnosis acute periprosthetic infection [19].

Initial intravenous antibiotics with Cefuroxime, Rifampicin and Piperacillin/Tazobactam was switched to Penicillin G in accordance with the antibiotic susceptibility testing. Intravenous antibiotics were administered for a total of four weeks. The patient was then discharged with oral antibiotics with Amoxicillin for a further four weeks. The further healing process was without complications. The patient died 18 months after the DAIR procedure due to heart failure, which was not related to the PJI.

**Fig. 1:**
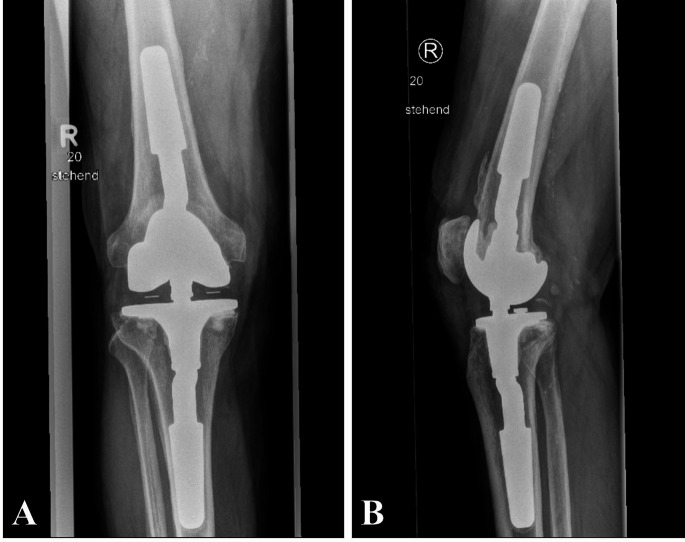
Preoperative X-ray images in two planes before DAIR Procedure (**A**: a.p.; **B**: lateral)

### Patient 2

**Fig. 2:**
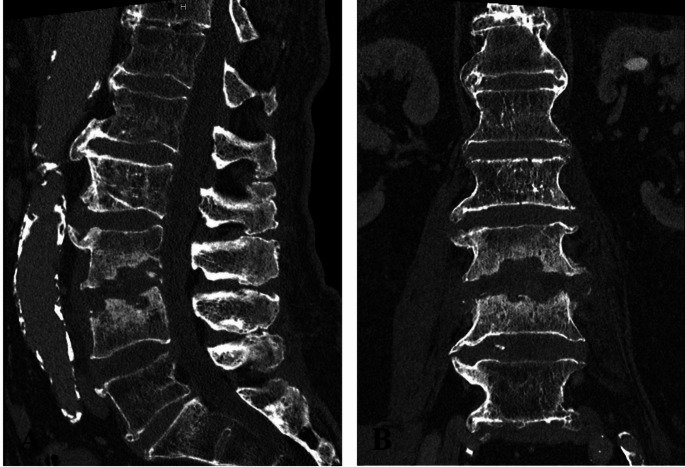
Preoperative CT-Images show destructive spondylodiscitis L3/L4 (A: sagittal plane; B: frontal plane)

**Fig. 3:**
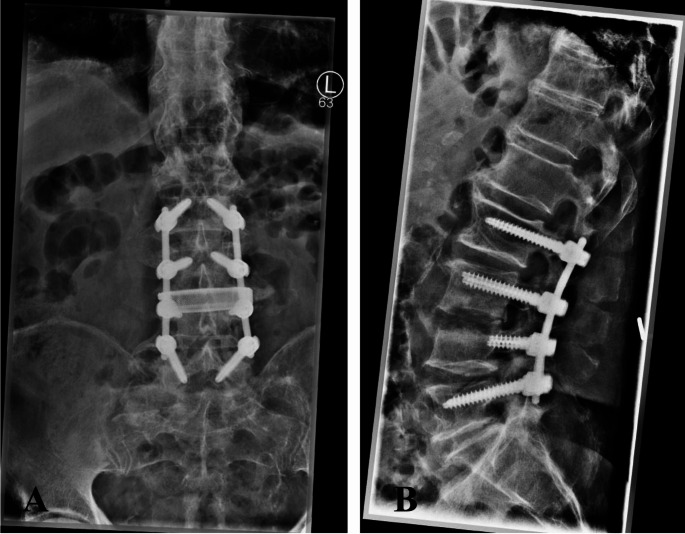
Postoperative radiological follow-up in two planes (**A**: a.p.; **B**: lateral) after two stage surgical treatment consisting of spondylodesis L2 to L5 with discectomy L3/4 and implantation of a XLIF

84-year-old male patient with pyogenic spondylodiscitis L3/4 with osseous destruction of the surrounding bone stock and associated lumbar pain with accompanying motor function impairment (slight weakness, 4/5 according to Janda, dorsiflexion of the right foot). In our emergency department, we took blood cultures before starting the intravenous antibiotic treatment with vancomycin. As a result of the advanced osseous destruction (Fig. 2), a two-stage surgical procedure was planned and performed (dorsal spondylodesis from L2 to L5 with decompression, debridement and irrigation of the disc space L3/4 followed by implantation of an extreme lateral interbody fusion (XLIF) cage L3/4 through right lumbotomy; Fig. 3).

In the preoperatively taken pair of blood cultures before start of the antibiotic treatment and surgery *S. infantarius* subspecies *coli* could be detected. Pathogen detection from intraoperative specimen was not possible. Postoperatively, the patient received intravenous antibiotic treatment with Vancomycin and Rifampicin for a total of three weeks in accordance with the resistogram. This was followed by oral antibiotic treatment with Amoxicillin and Clindamycin for a further three weeks. The further follow-up was without complications. At the last follow-up visit 15 months after surgery, the patient was fully recovered with no sign of a persisting infection.

### Patient 3

76-year-old female patient presented in 2019 at our hospital after a complex surgical history at several hospitals due to increasing pain in her right leg and a fistula at her right hip, that had closed spontaneously two months ago.

Initially, a primary right hip prosthesis had been implanted in 2004. After multiple soft tissue revisions, a revision total hip arthroplasty with implantation of a total femur replacement and was performed in 2014. In 2016, the patient was suffering under a chronic infection with *Pseudomonas aeruginosa*. Patient refused explantation, therefore a fistula was established. After spontaneous closure of the fistula in 2018, revision surgery was performed to create a new fistula. The samples obtained intraoperatively showed renewed evidence of *Pseudomonas aeruginosa* like the previous findings.

After about six months/in 2019, the patient presented again with a red, swollen leg with massive putrid fluid discharging from the existing. Subsequently, an explantation was performed.

The intraoperative tissue samples taken (six in total) showed a polymicrobial infection with detection of *P. aeruginosa*, *Staphylococcus aureus* and *Streptococcus mitis/oralis* in six out of six intraoperative specimen and *S. infantarius* subspecies *coli* from the sonication. We performed a targeted antibiotic therapy with (Amipicillin/Sulbactam and Ceftazidim). The histopathological examination revealed a type III membrane according to Krenn and Morawietz. Because of a complicated wound healing disorder, a total of four subsequent wound revisions had to be carried out, including the use of negative pressure wound therapy before final closure was possible. A renewed revision total hip arthroplasty is not planned. In the 60-month follow-up, the infection was completely cured in the existing resection arthroplasty. The patient is currently wheel-chair-bound due to handling problems with the pelvic orthosis.

### Patient 4

In 2024, a 79-year-old male patient presented via our emergency department due to lumbago and increased laboratory chemical infection parameters. The patient was previously treated in 2023 due to a spondylodiscitis L4/5 and L5/S1 with an epidural abscess and concomitant pneumonia. The patient underwent surgery (dorsal spondylodesis from L4 to S1 with a hemilaminectomy with decompression and disc evacuation of L4/5 and L5/S1 as well as a transforaminal lumbar interbody fusion (TLIF) cage implantation from the right into L4/5) and intravenous antibiotic treatment in 2023.

In 2024, the patient presented in our emergency department with impairment of his general condition. Further diagnostics revealed *Pseudomonas aeruginosa* from urine samples, which was treated with piperacillin/tazobactam intravenously. In addition, *S. infantarius* was detected in blood cultures taken from a central venous catheter. Subsequent Gadolinium-enhanced MRI imaging could exclude a renewed/persisting spondylodiscitis. Nevertheless, there was evidence for a deep soft tissue infection of the former surgical approach in the MRI-imaging. The patient was treated with intravenous antibiotics (penicillin G to address the *S. infantarius*) for a total of four weeks perioperatively and discharged with oral antibiotics (amoxicillin) for a further six months with significantly improved symptoms. At the follow-up visit 6 months after, the patient was fully recovered and very satisfied with the treatment result. Patient died 10 months after surgery because of a myocardial infraction.

## Discussion

This case series aims to enhance awareness for streptococcal musculoskeletal infections cause by *S. infantarius*. According to our knowledge, there have been no previous studies evaluating to role of *S. infantarius* in musculoskeletal infections.

Although treatment of all patients with musculoskeletal infection was successful, there is a need for further data to optimize treatment.

Overall, there are at the moment several studies describing methods such as genome typing or focus on the microbiological characterization of the pathogen [20, 21]. Although in general relevant, they do not focus on clinical aspects and do not aim to address health care professionals.

Kaindi et al. could outline in this context the role of *S. infantarius* as cause for bacteraemia, gastrointestinal tract infections, endocarditis while describing an association with colorectal cancer [6]. Whereas Counihan-Edgar et al. could describe cases of endocarditis in sea otters caused by *S. infantarius*. In contrast, endocarditis caused by *S. infantarius* in humans has not been reported yet [20]. Although we focused on musculoskeletal infections, we were able to identify a case of endocarditis caused by *S. infantarius*, with need for surgical therapy (valve replacement) in addition to antibiotic therapy, in our database. Furthermore, we were able to detect further eleven cases of bacteraemia with detection of *S. infantarius* in blood culture samples in our microbiological database. Against this background, the presented cohort is, to our knowledge, the largest described in the literature.

Musculoskeletal infections caused by *S. infantarius* have not been described in literature. Therefore, the presented cases might be very interesting for health care professionals. The four musculoskeletal infections that we were able to identify display the potential of infections with *S. infantarius* ranging from PJIs (*n* = 2), spondylodiscitis (*n* = 1), to postoperative wound infection after surgically treated spondylodiscitis (*n* = 1). In general, PJIs and spondylodiscitis are two of the most challenging infections which usually need complex surgical and long-term antimicrobial treatment [22]. In our cases, three out of four patients needed surgical treatment, and all our patients received at least six weeks of antibiotic treatment. As this is the first description of musculoskeletal infections caused by *S. infantarius* we cannot compare our data to literature.

In contrast, *S. infantarius* has been described as pathogen associated with gastroenterological or abdominal infections [23]. Corredoira et al. describe *S. infantarius* as causative pathogen in biliary tract infections able to cause bacteriemia. The relevant role in gastroenterological or abdominal infections is supported by our data (Table 1; *n* = 16/33) as 48.5% of all cases with an infection caused by *S. infantarius* are in the gastrointestinal and/or biliary tract.

In contrast only 12.1% (*n* = 4/33) cases involved the musculoskeletal system. This outlines that musculoskeletal infections with *S. infantarius* are still rare. A fact with is underlined by the low number of only 33 cases with an infection of *S. infantarius* over a period of 10 years. Here we must admit that despite all advances made in modern medicine we cannot rule out that some isolates might be identified as *S. bovis*.

*S. infantarius* and *S. bovis* are partly opportunistic germs that are known from the gastrointestinal tract of humans, animals and especially ruminants [5, 24].

*S. infantarius*, for example, has already been described several times in the literature in connection with fermented dairy products in Africa [8]. In addition, not every detection of *S. infantarius* is associated with a symptomatic infection. Fluid aspirations, tissue samples and sonication, for example, have a sensitivity ranging from 66,7% – 88,9% and a specificity ranging from 61,5% – 84,6% [25]. An aspect supported by our data as the detection of *S. infantarius* was found several times as an incidental finding in biopsies of the gastrointestinal tract (e.g. from bile secretions) without therapeutic relevance. In contrast, detection of *S. infantarius* from intraoperative samples or preoperative fluid combined with clinical symptoms of an infection necessitates subsequent therapy. The presented cases therefore outline the significance for an interdisciplinary approach.

To avoid infections the underlying mechanism of infection should be considered. Here, periprosthetic joint infections and vertebral osteomyelitis are often caused by pathogens of the skin flora e.g. as early or late infections in the context of intraoperative contamination [26, 27].

However, as *S. infantarius* is not known to be a commensal of the skin flora, this suggests that the infection is most likely to be haematogenous. Since *S. infantarius* has been described particularly in ruminants or in the context of African dairy products. The question of individual diets or nutritional habits of the treated patients would also be of interest to identify a possible causal chain [8, 24]. Nevertheless, none of the four patients with a musculoskeletal infection had such a history.

For successful therapy of the infection, knowledge of the antibiotic susceptibility pattern is essential. According to our knowledge, no antibiotic resistance has been described for *S. infantarius* in the current literature. We treated only two of our four orthopaedic patients with only penicillin. In the remaining two cases a polymicrobial infection was detected, therefore we could not limit the antibiotic treatment to penicillin. In the other case, due to the underlying severity of the disease we opted for continued vancomycin as an empirical treatment for a suspected methicillin-resistant *Staphylococcus aureus* infection.

As *S. infantarius* is no common pathogen for infection empiric therapy is often started with “broader spectrum” antibiotics (such as cefuroxim or in septic patients with vancomycin) and afterwards changed to penicillin according to resistogram.

As shown in Tables 3 and 100% of the detected isolates of *S. infantarius* were susceptible to penicillin, so that penicillin remains a valid therapeutic option for infections with *S. infantarius*. In the presence of a penicillin allergy, which is reported in general by about 10–15% of the hospitalized patients, the question of an alternative antibiotic therapy arises [28]. Our data show that clindamycin (85.7% susceptible isolates) as well as vancomycin (100% susceptible isolates) might be a valid alternative. Especially, clindamycin might be chosen for oral antibiotic therapy, like we used it in two of the presented cases. Comparative data are available from Corredoira et al., where all isolates were susceptible to penicillin, ceftriaxone and vancomycin. Corredoira et al. evaluated a 69% and 75% susceptibility rate of the *S. bovis* group (SBG) pathogens to clindamycin and levofloxacin, respectively. This indicates that *S. infantarius* may have a higher susceptibility towards clindamycin compared to the broader group of all SBG pathogens [23]. In addition, we could not find a single cases with a persisting infection after antibiotic therapy and need for a renewed stationary treatment according to our database.

Our study has some limitations. First of all, we were not able to access previous microbiological results of foreign laboratories or the exact history of the previously performed antibiotic therapies. In addition, we did not have access to the out clinic follow-ups of patients with non-musculoskeletal infections. Therefore, there might be a collection and selection bias. Moreover, the data were collected at a university hospital, where patients are often transferred to in case of complications or need for specialized treatment. In addition, identification of *S. infantarius* is a topic of ongoing debate as there is no gold-standard. The used system (Vitek-MS MALDI TOF) is according to Putham et al. non-inferior to 16 S rDNA and *sodA* gene sequencing [29].

In the initial treatment of an acute complex periprosthetic infection or spondylodiscitis, however, penicillin and clindamycin nevertheless may play a subordinate role, as a germ detection with a corresponding resistogram is usually only available a few days after sampling. Often broad-spectrum antibiotics are initially administered to address the most frequent pathogens, which cover *S. infantarius* although an infection with *S. infantarius* is not suspected.

## Conclusion

This study describes musculoskeletal infections caused by *S. infantarius*, highlighting its possible relevance as pathogen in orthopaedic infections. The findings underscore the importance of recognizing and appropriately treating *S. infantarius*. In case of penicillin allergy, clindamycin shows to be an effective alternative treatment.

## Data Availability

No datasets were generated or analysed during the current study.
